# Trials and tribulations of clinical research teaching and training

**DOI:** 10.4103/2229-3485.71773

**Published:** 2010

**Authors:** Ravindra B. Ghooi

**Affiliations:** *Bilcare Research Academy, 1002, Sai Capital, Senapati Bapat Marg, Pune – 411 016, India*

**Keywords:** Attrition, education, placements, research professionals, training

## Abstract

Clinical research institutions have mushroomed in the country, though there is a generalized lack of experienced faculty. These institutes mostly confine themselves to theoretical aspects of clinical research, since there is lack of facilities for practical training. Students passing out of these institutes often find it difficult to get decent jobs and salaries at the entry level in the industry are poor. Poor placements of graduating students become major barriers for attracting quality students to the courses. This in turn affects the quality of people that the industry requires, in order to ensure a high growth rate of the industry. The industry, in addition to facing a severe crunch of high quality professionals, is also suffering from attrition that is a common feature. This attrition stems from, inter alia the industry’s demand for experienced people at the entry level. To improve overall standards of professionals entering clinical research, institutes and the industry need to get together and work in close co-operation. The industry and the institutes need to take positive steps if recent trends have to be reversed and clinical research as a whole has to move to a higher level. This article is based on the perceptions of the author, about the problems faced and offers some suggestions. Though these perceptions represent the reality, it is difficult to provide hard evidence that they do so.

## CLINICAL RESEARCH TEACHING AND TRAINING

Clinical research activities have stepped up in the last one or two decades. Drug discovery and development activities in the country have accelerated following a change in the patent laws; additionally western countries are outsourcing more research work to countries like India and China. Industry observers have identified numerous factors that drive the outsourcing process for research, which include good medical facilities, well trained investigators, adequate patient population and lower costs of research. The Indian Government has given a fillip to clinical research by extending custom duty exemption for imports and waiving of service tax. It is widely acknowledged that there is still something lacking, and when viewed from the perspective of someone who has worked both with the industry and the academics, some issues become pain points. It is acknowledged that hard evidence is lacking, and these are perceptions. Yet these have emerged through years of deep involvement, and may help in providing solutions to the problems perceived.

Financial consultants McKinsey predicted a tremendous employment potential, as early as in 2004 in this sunrise industry. Similar forecasts were made by the Boston Consulting Group, with the report “Looking Eastwards” in 2006[1] and the Ernst and Young with the report “The Glorious Metamorphosis - Compelling Reasons for doing Clinical Research in India” in 2009.[2] These highly respected organizations strengthened the main prediction attributed to McKinsey, that India would be doing between 1500 and 2000 GCP trials in 2010. The National Institutes of Health (NIH) website shows that as of October 8, 2010 there are 1639 trials in progress in India, which vindicates predictions by these financial consultants.

The lack of high quality qualified and trained support staff remains a constraint and if not addressed, could impede the growth of this industry. Clinical research is not taught in medical, pharmacy or science colleges, though now some parts of it are being introduced in the syllabi. The need for specialized training in this field was felt as early as in 2004 and the first Institute devoted to training graduates in clinical research was set up. Institute of Clinical Research India (ICRI) set up its first campus in Dehradun (subsequently this was shifted to Delhi), and other campuses were set up in Mumbai, Bangalore and Ahmedabad. Bilcare Research Academy set up its first campus in Pune in 2007. Clinical Research Education and Management Academy (CREMA) set up its campuses in Delhi, Mumbai, Bangalore, and Hyderabad between 2007 and 2009. Additionally, a large number of private players entered this field and now there are clinical research institutes in many cities and towns.

With graduates of many institutes now available for placement, one would have expected that the shortage of support staff would have been met with. However, it appears that the industry is not satisfied with the output from these institutes, as is evidenced by the falling numbers of students placed, the falling salaries that are offered and the large number of vacancies that remain unfilled in the industry. These are concerns both for the institutes and the industry and need to be examined in depth, and solved. A common comment of the industry has been that students from clinical research institutes have only theoretical knowledge and they lack any experience in actually conducting research.

## INDUSTRY’S EXPECTATION

In the past, the research industry picked up graduates from medical and pharmacy colleges and trained them as per their requirements. When graduates with qualifications in clinical research became available, they were preferred over doctors and pharmacists who had no exposure to clinical research. The industry then wanted students who had practical in addition to theoretical knowledge. Many companies started asking for candidates with experience even for entry level posts, while salaries remained more of less static.

## INSTITUTES’ PROBLEMS

Most institutes are privately funded, and depended on the fees received from students for their continuance. They cannot afford to be choosy with the quality of students seeking admission. With falling salaries and placement, the quality of students falls further. These institutes are not affiliated to hospitals, and hence giving practical training to their students is not possible. When clinical research industry was approached for internship of students, they cited confidentiality as an issue that prevented taking on interns. Medical graduates did get internship in hospitals, but they were put on routine patient care duties and hence the internship did not help.

Though the spread of education in the country is appreciable, there is a continual fall in the quality of students passing out of colleges. In addition to poor grasp of basic scientific concepts, their command over spoken and written English is exasperating. Increasing language chauvinism has further eroded the capabilities of graduates while communicating in English. This has led to a fall in quality of students joining clinical research institutes. Experience of trying to include basic English in their curricula has not been satisfactory. In the short one year that the students are with these institutions, it is difficult to undo the harm done in the past decades by bad teachers.

## STUDENT’S PERSPECTIVES

The student coming for admission to a vocational course like clinical research has only one objective, and that is good placement. Most consider education only as a means for earning a good salary, and the bench mark is the IT industry. The quality of students is directly proportional to the average salary at entry level on completion of the course, and as this drops, so does the quality. This reflects on the quality of output from the institutes.

Additionally the output depends on other factors like the quality of faculty, the syllabus and the exposure to industry.

## QUALITY OF THE FACULTY

Attracting teachers who have industry experience to teach in an institute is a major problem for most institutes. One of the main reasons is that the compensation in the institutes is too low in comparison with that in the industry. Institutes based in Mumbai or Bangalore are probably better off, since the industry is concentrated there. For institutes are based out of the industrial hub, the difficulties are almost insurmountable. As a result, very few institutes have staff that is competent to teach clinical research, and many institutes operate without a full complement of staff.

Most senior faculty members have come from the pharmaceutical industry, having made a lateral shift to research. They do not have professional qualification in the subjects they teach and hence the quality of teaching is not uniform. In the absence of a professional qualification, approval from a professional body will ensure a minimum standard for the faculty. There needs to be a ‘trainer approval’ program for faculty.

## SYLLABUS

The universities to which most institutes are affiliated do not have a clinical research department; as a result, the affiliation does not add value to the course. The university rarely has a say in the syllabus, and even if it were to have a say, its expertise is doubtful. Affiliation with a professional body would be an obvious advantage in setting up a syllabus which meets with the professional body’s approval.

It would be difficult to comment on the syllabi presently taught by different institutions. From the point of view of what is practically required by clinical research associates (CRA) and clinical research coordinators (CRC) a model syllabus could be built, and members of the Indian society for Clinical Research invited to comment on it, thus helping the syllabus to evolve into a need based one.

## INDUSTRY EXPOSURE

Exposure of students to the industry requires cooperation of the industry, which is sadly lacking. There is a lot of discussion on academia industry interface in all spheres of education, yet on the ground level, the interface is all but non-existent. Most industry personnel forget that they too had little or no knowledge about clinical research, when they joined in the industry, yet they expect fresh students to have much more knowledge.

The supply of trained personnel from clinical research institutes and their employment by sponsors and CROs follows the laws of supply and demand. The industry is saving a considerable amount of money by employing people who are at least partly trained by institutes. We admit that there are lacunae in the training programs, but these can only be removed if the industry cooperates with the institutes. It might therefore be germane to examine the role that the industry plays in this.

## SALARIES IN THE INDUSTRY

The falling salaries in the industry have already been touched upon, yet there is need to examine the issue more thoroughly. Barring a few organizations, the payment to clinical research professions at entry level is pathetic. It must be pointed out that lowest minimum wage in the state of Maharashtra is that for skilled workers in vita and kaule (brick and roof) tiles manufacturing industry, and that is Rs. 153.46 per day. This works out to Rs. 4603 per month.

Compare this with a monthly package of Rs. 2500 offered by a CRO for a graduate student with a post graduate degree in clinical research. Another pharmaceutical company offered Rs. 4000 per month to students, which the institute turned down. The employers were smart enough to call this a stipend, so as not to fall foul with the government regulations. But the message is clear; employers are not willing to pay clinical research professionals as much as they pay their lowest paid employees.

Low salaries bring disrepute to the research industry; they lower the morale of the people and send a very clear negative signal to prospective students. Because of such policies, the best student material is not attracted toward clinical research, and the quality of students joining research institutes drops further.

## ATTRITION

The attrition rate is big issue for the industry. Low salaries and lack of increments that are offered are a common cause of attrition. When salaries paid are so low, that the person cannot even survive in the city, the person will spend most time searching for another job. Only when the employee puts in his papers, he or she is told that the management was considering the individual for a promotion. Undoubtedly, many readers have faced this, during their own careers.

Unlike what most people believe, attrition is not solely due to the employee’s attraction for money. Attrition is due to both employee-related and employer-related factors. Money is not the only factor for which a person leaves his or her job, quite often it is due to the employee being uncomfortable with seniors. ‘People leave their immediate managers, not the companies they work for’,[3] is as applicable here as elsewhere.

An important employer-related issue which is the major cause of attrition is the demand for experienced people, even at entry level. When one company wants to employ only experienced persons, poaching from another company is inevitable. What is talent search for one employer is attrition for another.[4]

Many people in the pharmaceutical and clinical research industries were denied increments in 2009 and 2010, in the name of recession. IMS data tells us, that the pharmaceutical sales in India were not affected by recession,[5] but there is no reliable data for clinical research turnover available, hence the true impact of recession is unknown.

## ROLE OF THE ACADEMIA

Clinical research institutes have mushroomed all over the country; these institutes are staffed by people who have little or no exposure to clinical research. Faculty that teaches in many of these institutes would not pass a CRA or CRC examination. Very often, general practitioners are invited to lecture, in the hope that one who can treat patients should be able to teach research.

Many of these institutes have started course for specialization in areas like clinical data management, quality assurance, and business development etc. While there is no disputing the need for specialization, one wonders what the syllabus of these courses is. The fact that there is no national body overseeing specialized education in the country is exploited widely. This is not to recommend oversight of clinical research education by a body like the AICTE or Medical Council of India (the less said about these bodies, the better), but some sort of regulation is required.

Clinical research institutes need to get their act together, and stop viewing students as a source of income. They should keep the syllabus updated and interact with the industry to develop students as the industry wants. Obviously this is not going to be achieved by the institutes on their own; it will need some effort on the part of the industry too.

## INDUSTRY ACADEMIA INTERFACE

Most institutes invite industry experts to come and address their students. Experience has taught us that these experts are willing to come only on Saturdays or Sundays, and demand to be paid at rates, way above what the institutes can afford. They are not willing to speak on difficult topics, which the institutes want them to speak on. The favourite topic for industry experts is “Good Clinical Practices (GCPs)”. For the institute this raises another problem, how many lectures can one have on GCP?

Another area of disappointment for the students is the trial work in progress in the country. Whenever a student asks the visiting industry experts about the trials their company is presently conducting, the reply is ‘that is confidential’. The fact that details of trials in India should be available on the registry (www.ctri.in) is often forgotten both by the industry experts and by our students. This response does not show the industry’s concern for confidentiality, but it rather shows their unwillingness to share any information with students.

## THE WAY FORWARD

For the health of clinical research in India, both the institutes and the industry must survive. They are dependent on each other with their success and failure is linked. For a healthy growth of clinical research, industry and academia should come together and help each other grow. Without active support of the industry the institutions will not survive, and even if they do, they will not be able to put out students who the industry wants.

From the academia’s side, they must ensure high quality teaching and training. Students should be enrolled with a view to maximize the quality of graduates in this space, and not to maximize profits. Institutes should provide courses for specialization in areas such as regulatory affairs, clinical data management, business development rather than a single course in clinical research. The financial viability of such specialized courses needs to be worked out.

It is pity that in many instances academic institutes are called business units. The minute education is treated as business, the sanctity of education is lost, it becomes yet another profit-oriented activity.
